# *Synechococcus elongatus* UTEX 2973, a fast growing cyanobacterial chassis for biosynthesis using light and CO_2_

**DOI:** 10.1038/srep08132

**Published:** 2015-01-30

**Authors:** Jingjie Yu, Michelle Liberton, Paul F. Cliften, Richard D. Head, Jon M. Jacobs, Richard D. Smith, David W. Koppenaal, Jerry J. Brand, Himadri B. Pakrasi

**Affiliations:** 1 Department of Biology, Washington University, St. Louis, MO 63130; 2 Genome Technology Access Center, Washington University School of Medicine, St. Louis, MO 63110; 3 Pacific Northwest National Laboratory, Richland, WA 99352; 4 UTEX The Culture Collection of Algae, University of Texas at Austin, TX 78712

## Abstract

Photosynthetic microbes are of emerging interest as production organisms in biotechnology because they can grow autotrophically using sunlight, an abundant energy source, and CO_2_, a greenhouse gas. Important traits for such microbes are fast growth and amenability to genetic manipulation. Here we describe *Synechococcus* *elongatus* UTEX 2973, a unicellular cyanobacterium capable of rapid autotrophic growth, comparable to heterotrophic industrial hosts such as yeast. *Synechococcus* UTEX 2973 can be readily transformed for facile generation of desired knockout and knock-in mutations. Genome sequencing coupled with global proteomics studies revealed that *Synechococcus* UTEX 2973 is a close relative of the widely studied cyanobacterium *Synechococcus* *elongatus* PCC 7942, an organism that grows more than two times slower. A small number of nucleotide changes are the only significant differences between the genomes of these two cyanobacterial strains. Thus, our study has unraveled genetic determinants necessary for rapid growth of cyanobacterial strains of significant industrial potential.

Among the photosynthetic organisms, cyanobacteria offer attractive systems for biotechnological applications due to their increased growth rate compared to plants and their relative ease of genetic manipulation compared to eukaryotic algae^1^,^2^,^3^. Unlike heterotrophic microbes such as the bacterium *Escherichia coli* and the yeast *Saccharomyces cerevisiae* that are of common industrial use, cyanobacteria can grow photoautotrophically by harvesting light energy. The ability to use solar energy to fix carbon dioxide and generate oxygen has made cyanobacteria one of the primary producers on our planet^4^. There is considerable current interest in using cyanobacteria to mitigate the effects of increasing amounts of atmospheric carbon dioxide^5^. Some strains of cyanobacteria can accumulate large amounts of lipids and are excellent candidates for biofuel production^3^. Also, cyanobacterial biomass is becoming a compelling alternative to biomass from food crops in the bioenergy arena^6^. Cyanobacteria can grow in fresh or seawater, or even wastewater, thus avoiding the potential conflict between food and fuel production. Furthermore, cyanobacteria have a broad spectrum of secondary metabolites, including amino acids, fatty acids, macrolides, lipopeptides, amides^7^, some of which are potential pharmaceutical agents. Recently, three cyanobacterial model strains, *Synechocystis* sp. PCC 6803, *Synechococcus* *elongatus* sp. PCC 7942 and *Synechococcus* sp. PCC 7002, have been used in synthetic biology studies for biosynthesis of multiple products including free fatty acids^8^, isoprene^9^, 2,3-butanediol^10^, 1-butanol^11^, squalene^12^, *n*-alkanes^13^ and hydrogen^14^.

Despite these advantages of using cyanobacteria in biotechnological settings, some limitations have kept these organisms from becoming the preferred microbial platforms in such applications. So far, the genetic and metabolic networks in cyanobacteria are not as well understood as in microbes such as *E. coli* and *S. cerevisiae*, and the biological “parts list” in cyanobacterial cells are not standardized and well-characterized^2^. In contrast, the *E. coli* system has been extensively studied, so that metabolic networks are known in detail and a wide array of synthetic biology tools are available. However, phototrophic cyanobacterial cells are functionally quite distinct from heterotrophic *E. coli* cells^15^, resulting in the limited application of current biotechnological tools, which cannot be directly applied to cyanobacteria without modification. Efforts focusing on understanding cyanobacterial systems are underway; however, one drawback compared to *E. coli* or yeast is that the growth rates of commonly used cyanobacterial model strains are significantly slower, requiring extended timeframes (weeks to months) to accomplish synthetic biology experiments that can be performed in *E. coli* or yeast in days. Also, many cyanobacteria contain multiple copies of chromosomes (~12 copies/cell in *Synechocystis* PCC 6803^16^ and ~ 5–7 copies/cell in *Synechococcus* PCC 7942^17^). Therefore, eliminating a gene from all copies of the chromosome may take several rounds of segregation.

A cyanobacterial strain that can grow rapidly and is amenable to facile, targeted genetic manipulation would expedite the process of standardizing and characterizing biological parts of cyanobacterial systems. We therefore sought to identify and characterize such a strain in order to build a superior cyanobacterial chassis for synthetic biology and metabolic engineering applications. In this communication, we describe the identification of a unicellular cyanobacterium *Synechococcus elongatus* UTEX 2973 (hereafter *Synechococcus* UTEX 2973), a relative of *Synechococcus elongatus* PCC 6301 and PCC 7942, that displays a substantially faster growth rate than any other commonly used cyanobacterium. We determined the genome sequence of *Synechococcus* UTEX 2973 and analyzed its global proteome. Furthermore, we determined that targeted genetic modifications can be rapidly engineered in this organism. Our data demonstrate the excellent potential of *Synechococcus* UTEX 2973 as a cyanobacterial chassis for broad biotechnological applications.

## Results

### Rapid growth of *Synechococcus elongatus* UTEX 2973

**Figure 1:**
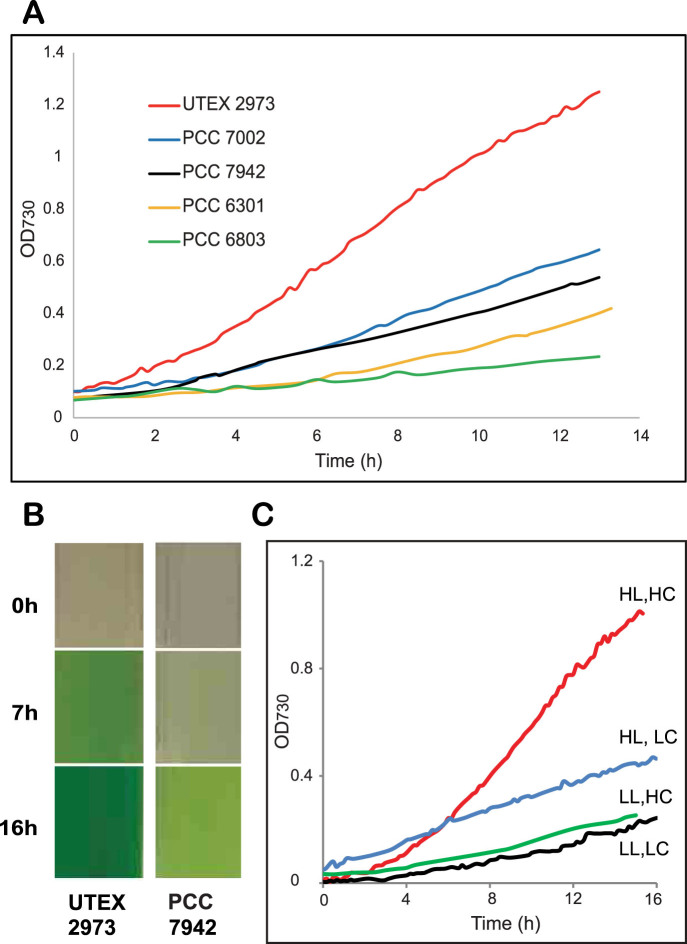
Comparison of growth rates of different cyanobacterial strains. (A) Growth curves of strains under individual optimal conditions. *Synechococcus* UTEX 2973: 41°C, 500 µmole photons·m^−2^·s^−1^; *Synechococcus* PCC 7002: 38°C, 500 µmole photons·m^−2^·s^−1^; *Synechococcus* PCC 7942: 38°C, 300 µmole photons·m^−2^·s^−1^; *Synechococcus* PCC 6301: 38°C, 500 µmole photons·m^−2^·s^−1^; *Synechocystis* PCC 6803: 30°C, 300 µmole photons·m^−2^·s^−1^. All cultures were grown with 3% (v/v) CO_2_ bubbling. (B) Visual comparison of culture densities of *Synechococcus* UTEX 2973 and *Synechococcus* PCC 7942 during a 16 h growth period. (C) Growth curves of *Synechococcus* UTEX 2973 at 38°C under different conditions. HL (high light): 500 µmole photons·m^−2^·s^−1^; LL (low light): 100 µmole photons·m^−2^·s^−1^; HC (high CO_2_, 3%); LC (low CO_2, _0.04%). Measurements were made every 10 min. Five replicate growth curves were generated for each strain under indicated conditions, except for *Synechocystis* PCC 6803, which had two replicates. The representative curves are shown in the figure.

In 1955, Kratz and Myers^18^ described a fast growing cyanobacterial strain, *Anacystis nidulans*. This strain was subsequently deposited in the University of Texas algae culture collection as *Synechococcus leopoliensis* UTEX 625. However, during recent years, this strain lost its rapid growth property (see Methods section) and was also unable to grow at a temperature as high as 38°C, unlike the original strain described by Kratz and Myers^18^. We selected a single cyanobacterial colony from a mixed culture of *Synechococcus* UTEX 625 that was able to grow at 38°C, and deposited the resulting strain to the UTEX algae culture collection as *Synechococcus elongatus* UTEX 2973. Growth of *Synechococcus* UTEX 2973 was assessed under different conditions and the shortest doubling time was 1.9 hrs in BG11 medium^19^ at 41°C under continuous 500 μmoles photons·m^−2^·s^−1^ white light with 3% CO_2_. This is a remarkably high growth rate under autotrophic condition, the highest rate reported to date for a cyanobacterial strain, and comparable to heterotrophic growth rates of the yeast *S. cerevisiae*. The doubling time of *Synechococcus* UTEX 2973 increased to 2.3 hrs at 38°C (Table 1). However, this rate is still nearly twice as fast as that for *Synechococcus* PCC 7942 (~4.1 hrs) under similar conditions (Fig. 1A). Growth rates of three other commonly used cyanobacterial strains, *Synechocystis* sp. PCC 6803, *Synechococcus* sp. PCC 7002 and *Synechococcus* PCC 6301, were also significantly slower than that of *Synechococcus* UTEX 2973 (Fig. 1A and Table 1). A direct result of such a rapid growth property is that a dilute culture of *Synechococcus* UTEX 2973 accumulated substantial amounts of biomass within a 16 h time period. In contrast, a *Synechococcus* PCC 7942 culture was still dilute when both cultures were grown under their respective optimal conditions (Fig. 1B). Similar results were obtained for biomass accumulation of these two strains. The dry weight of *Synechococcus* UTEX 2973 increased from 0.13 ± 0.01 mg/ml at the beginning of the experiments to 0.87 ± 0.03 mg/ml after 16 hours, while the dry weight of *Synechococcus* PCC 7942 changed from 0.12 ± 0.02 mg/ml to 0.33 ± 0.03 mg/ml during the same period of time. As shown in Fig. 1C, a high level of illumination as well as CO_2_ are important factors to support rapid growth of *Synechococcus* UTEX 2973 (Fig. 1C).

### Genomic and Proteomic analysis of *Synechococcus* UTEX 2973

**Figure 2:**
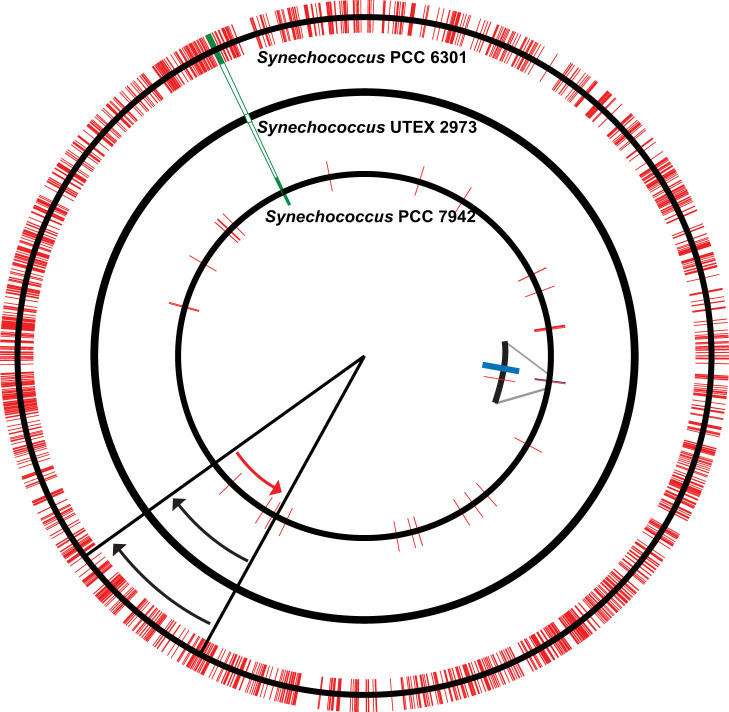
Circular maps comparing related cyanobacterial genomes. Middle circle, *Synechococcus* UTEX 2973; outer circle, *Synechococcus* PCC 6301; inner circle, *Synechococcus* PCC 7942. (red bar), single nucleotide substitutions and indels compared to *Synechococcus* UTEX 2973; (green box), deletion; (blue box), insertion. Red and black arrows indicate the opposing orientations of the inverted region.

The original *Anacystis nidulans* strain^18^ was also deposited to the Pasteur Culture Collection as *Synechococcus* sp. PCC 6301. Whole genome sequencing of *Synechococcus* UTEX 2973 indicated that this strain is similar to *Synechococcus* PCC 6301 in terms of the general features of their genomes (Table 2). Unexpectedly, the genome sequence of *Synechococcus* UTEX 2973 exhibited remarkable similarity to that of the widely studied model cyanobacterium *Synechococcus* PCC 7942, an organism that has apparently been isolated from a distant geographical location. In fact, there were a total of 55 single nucleotide polymorphisms (SNPs) and insertion-deletions (indels) between the two genomes. Among these 55 differences, 39 were in chromosomes and 16 were in plasmids. Among the 39 differences in chromosomes, 28 were in protein coding regions, with 26 of these shown in Table 3, plus a large deletion shown in Table S1 and a 188.6 kb inversion shown in Fig 2 and Table S2. Among the 16 differences in plasmids, 7 were in protein coding regions (Table 4). We examined these differences and identified the affected genes, some of which are important components of known biochemical processes (Tables 3 and 4). For instance, the gene encoding the ATP synthase F_1_ subunit α in *Synechococcus* UTEX 2973 contains an SNP compared to the corresponding gene in *Synechococcus* PCC 7942. This SNP causes an amino acid substitution (Tyr in UTEX 2973 changed to Cys in PCC 7942) at the 252th position in this protein (Table 3). Importantly, detected peptides in our global proteomics study (see below) confirmed the Y to C replacement at that position in the two strains.

Surprisingly, the number of detected nucleotide differences between *Synechococcus* UTEX 2973 and PCC 7942 was orders of magnitude less than those between *Synechococcus* UTEX 2973 and *Synechococcus* PCC 6301 (~1600 SNPs and indels), the closest relative based upon strain history. Despite the fact that *Synechococcus* UTEX 2973 and PCC 7942 are 99.8% identical in their genome sequences, *Synechococcus* UTEX 2973 can grow more than twice as fast as *Synechococcus* PCC 7942. It is reasonable to hypothesize that one or more of the relatively small number of genes with the observed SNPs and indels determines how cells utilize available resources and thus controls the growth rate of these cyanobacterial cells.

In order to further compare these two strains at their proteome level, global proteomics analysis was performed for both cyanobacterial strains. In-depth LC-MS/MS proteomic analysis of *Synechococcus* UTEX 2973 identified peptides belonging to 1754 unique proteins (66% coverage), 1180 of which could be categorized into different functional groups (Fig. S1). As described above, *Synechococcus* UTEX 2973 and PCC 7942 are 99.8% identical in their genome sequences, so that most detected peptide sequences are indistinguishable between these two strains. However, due to the overall deep coverage, our MS data detected many of the amino acid changes that are predicted as a consequence of the SNPs between the two strains (Table 3 and 4).

A 5,764 bp sequence that contains six open reading frames in *Synechococcus* PCC 7942 was missing in *Synechococcus* UTEX 2973 (Fig. 2, Table S1). Although most of these genes do not have any annotated function, transcriptomics analysis (data not shown) indicated that these six genes are constitutively transcribed in *Synechococcus* PCC 7942, providing an intriguing avenue for further study. In addition, our proteomics study confirmed the presence of the protein products of five of these six genes in *Synechococcus* PCC 7942 (Table S1).

A previous study had indicated that there is a 188.6 kb inversion between the *Synechococcus* PCC 6301 and *Synechococcus* PCC 7942 genomes^20^. We determined that *Synechococcus* UTEX 2973 has the same genome arrangement in this inversion region as *Synechococcus* PCC 6301 (Fig. 2, Table S2). This inversion includes the coding region for a porin-like protein, and was proposed to be related to the natural competency (for DNA uptake) of these cyanobacterial cells^20^ (see below).

### Ultrastructural analysis of *Synechococcus* UTEX 2973

**Figure 3:**
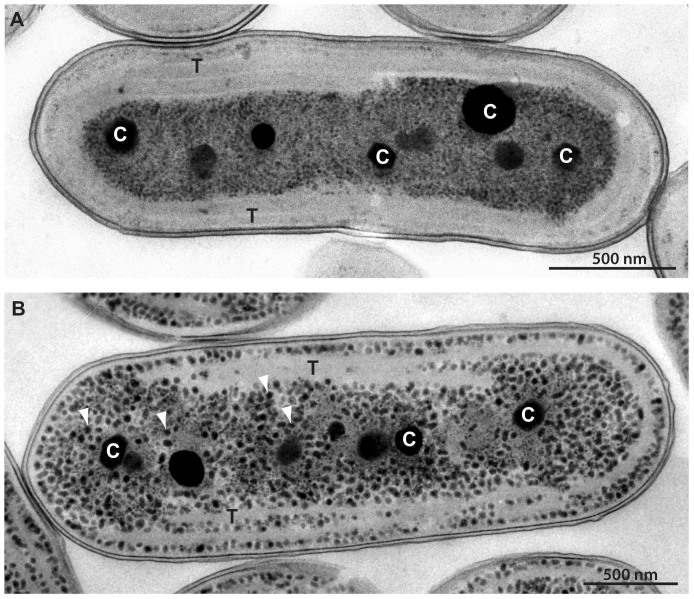
Ultrastructural comparison of *Synechococcus* strains. Electron micrographs of (A) *Synechococcus* UTEX 2973 and (B) *Synechococcus* PCC 7942 grown in 3% CO_2_. Labeled are carboxysomes (C) and thylakoid membranes (T). White arrowheads point to the numerous electron-dense bodies. Bar = 500 nm.

Cells of *Synechococcus* UTEX 2973 and *Synechococcus* PCC 7942 were examined by electron microscopy to identify structural differences between these strains at the subcellular level (Fig. 3). Both strains are rod-shaped cells typically > 2 µm in length. The size of the cells was similar between the two strains and did not appear to vary between growth in air and growth in 3% CO_2_. Intracellular organization was also similar between the strains when grown in air, with 2–3 thylakoid membrane layers forming evenly spaced concentric rings that followed the shape of the cell envelope. Carboxysomes and polyphosphate bodies were located in the central cytoplasmic region, and the number of these bodies was similar in the two strains. When grown in 3% CO_2_, the most striking difference between the two strains was the appearance of numerous electron-dense bodies in *Synechococcus* PCC 7942 (Fig. 3B). These bodies were found throughout the entire cell, including between and surrounding the thylakoid membrane layers, and coincided with a disorganized appearance of the thylakoid membranes and a decrease in the number of thylakoid layers to 1–2 in most cells. These electron-dense bodies were nearly completely absent in *Synechococcus* UTEX 2973 (Fig. 3A) when grown in 3% CO_2_. These bodies in *Synechococcus* 7942 are spherical or slightly elongated and approximately 30 nm in size. To our knowledge, the occurrence of such inclusions under high CO_2_ conditions has not been previously observed in *Synechococcus* strains.

### Genetic manipulation of *Synechococcus* UTEX 2973

**Figure 4:**
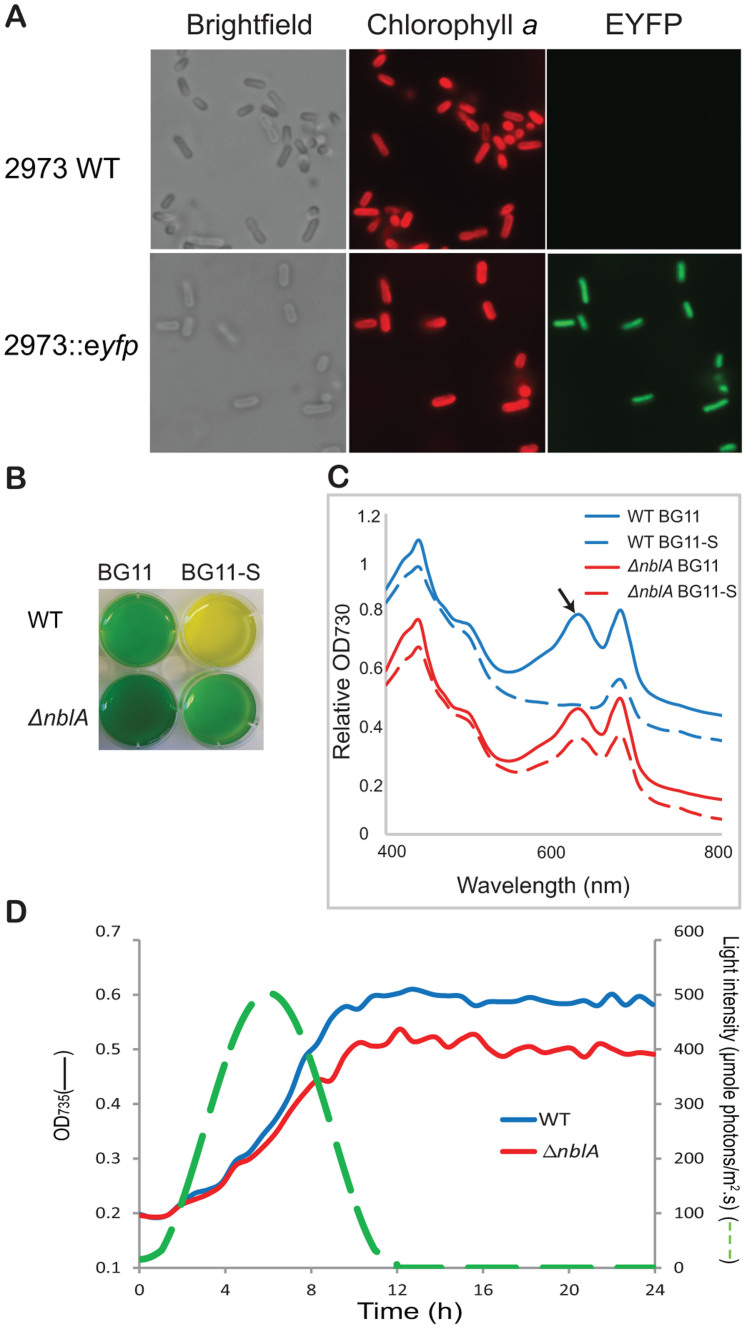
Directed gene manipulation in *Synechococcus* UTEX 2973. (A) Light micrographs of WT cells and EYFP-expressing transformants. Shown are brightfield (left column), chlorophyll *a* fluorescence (middle column), and EYFP fluorescence (right column) images. (B) Color phenotypes of WT and *ΔnblA* mutant cells. Under sulfur deprivation (BG11-S), WT cells bleached but Δ*nblA* cells did not. (C) Absorption spectra of WT (blue) and Δ*nblA* (red) cells. Solid lines, sulfur replete; dashed lines, sulfur deprived. The phycocyanin peak at 625 nm is designated by the arrow. Curves were vertically offset for clarity. (D) Growth curves of WT (blue) and *ΔnblA* (red) cultures under varying irradiance (green dashed line) to mimic a natural 12 h light/12 h dark cycle.

Amenability to facile genetic manipulation is a key requirement for a strain to be used as a chassis in synthetic biology and metabolic engineering applications. Three widely used cyanobacterial strains, *Synechocystis* 6803^21^, *Synechococcus* PCC 7942^22^,^23^ and *Synechococcus* PCC 7002^24^, are naturally competent, actively taking up exogenous DNA from their surroundings and incorporating it into their genomes. However, this feature is not common among bacterial strains, so that other widely studied cyanobacteria, including some nitrogen fixing strains of *Nostoc* and *Anabaena,* are routinely genetically manipulated through conjugative transfer of DNA from *E.* coli cells^25^. Previous studies of conjugative DNA transfer to cyanobacterial cells have established a well developed system with several conjugal, helper and suicide or replicable cargo plasmids available for use^26^. We determined that *Synechococcus* UTEX 2973 cells were not naturally competent, like its close relative *Synechococcus* PCC 6301. However, conjugation through tri-parental mating^22^ (also see Materials and Methods) was successfully applied to *Synechococcus* UTEX 2973 cells with a stable efficiency of ~1–2 × 10^−5^ colonies/plated cell. In order to clearly show the occurrence of transgene introduction using a replicable cargo plasmid or homologous recombination using a suicide cargo plasmid, mutants were generated with distinct phenotypes. As a transgene, the gene encoding an enhanced yellow fluorescent protein (EYFP) gene was introduced into *Synechococcus* UTEX 2973 cells on the self replicating vector pTrc-EYFP with expression of the transgene controlled by the Trc promoter^15^. Mutant cells exhibited strong fluorescence from YFP (Fig. 4A), so that the intensity of emitted fluorescence from EYFP-expressing mutant cells was about ~470 times higher than that from wild type (WT) cells.

To demonstrate targeted gene replacement in *Synechococcus* UTEX 2973, we inactivated the *nblA* gene. In other cyanobacteria, the *nblA* gene has been shown to function in pathways involved in regulation of light harvesting, and is particularly noted for its role in phycobilisome degradation under nutrient starvation conditions^27^. In *Synechococcus* PCC 7942, *nblA* transcripts accumulate to high levels when cells are starved for nitrogen or sulfur, and the resulting loss of phycobilisomes lends cultures a characteristic bleached color^28^. The Δ*nblA* mutant was engineered by homologous recombination of a suicide cargo plasmid with the *nblA* gene in the chromosome of *Synechococcus* UTEX 2973 (see Materials and Methods and Fig. S2). WT cells of *Synechococcus* UTEX 2973 turned yellow in BG11-S media within 7 days (Fig. 4B). Cells of the *Synechococcus* UTEX 2973 Δ*nblA* mutant, in which the *nblA* gene was completely deleted, still appeared blue-green in BG11-S after 7 days (Fig. 4B). Whole cell absorbance spectra (see Materials and Methods) showed that *Synechococcus* UTEX 2973 WT and Δ*nblA* mutant cells growing in regular BG11 medium had a similar ratio of phycobilin absorbance at 630 nm vs. chlorophyll *a* absorbance at 680 nm (~0.94) (Fig. 4C). This ratio decreased considerably (to ~ 0.7–0.8) when *Synechococcus* UTEX 2973 WT cells were grown without combined sulfur. However, this ratio did not significantly change in *Synechococcus* UTEX 2973 Δ*nblA* mutant cells grown under the same nutrient starvation conditions (Fig. 4C), confirming that the engineered Δ*nblA* mutant strain of *Synechococcus* UTEX 2973 had a similar phenotype as the Δ*nblA* mutant of *Synechococcus* PCC 7942^28^. Under nutrient replete condition, the Δ*nblA* mutant strain of *Synechococcus* UTEX 2973 showed similar growth rates compared to WT cells under constant light conditions, an observation similar to that of the *Synechococcus* PCC 7942 Δ*nblA* mutant strain^28^. Interestingly, the newly generated Δ*nblA* mutant strain exhibited slower growth compared to the WT *Synechococcus* UTEX 2973 strain when cells were grown under an illumination condition that mimicked a natural day-night cycle (see Methods section). This new finding indicates that the presence of the NblA protein provides a growth advantage to cyanobacterial cells under natural conditions.

## Discussion

A rapidly growing cyanobacterial strain that can be genetically manipulated would serve as an ideal candidate for broad research purposes, including studies that focus on understanding cyanobacterial systems and those that utilize cyanobacteria to produce valuable products. As mentioned earlier, none of the currently used model cyanobacterial strains can grow under autotrophic conditions as fast as yeast or other microbes used in industrial applications. In order to fill this gap, we looked for “new” cyanobacterial strains, and *Synechococcus* UTEX 2973 stood out as strain that grew especially rapidly. For example, *Synechococcus* UTEX 2973 grew faster than *Synechococcus* PCC 6301, *Synechococcus* PCC 7942, *Synechococcus* PCC 7002 and *Synechocystis* PCC 6803 (Fig. 1A). Surprisingly, *Synechococcus* PCC 6301 and *Synechococcus* UTEX 625 have been described as identical according to the Pasteur Culture Collection (PCC). However, our axenic cultures of *Synechococcus* UTEX 2973 behaved differently than cultures of *Synechococcus* PCC 6301. Whole genome sequencing confirmed that *Synechococcus* UTEX 2973 and *Synechococcus* PCC 6301 are definitely two uniquely different strains. Previously published data on the doubling time of *Synechococcus* PCC 6301^29^ as well as our current growth data (Table 1) suggest that the growth of *Synechococcus* PCC 6301 is significantly slower than the *Anacystis nidulans* strain that was isolated in 1952 and studied in 1955^18^. In contrast, growth of *Synechococcus* UTEX 2973 (this study) is similar to that of the original *Anacystis nidulans* strain^18^. An interesting observation from genome alignments (Fig. 2) is that the *Synechococcus* UTEX 2973 genome is more similar to the *Synechococcus* PCC 7942 genome, even though these two strains were isolated from different geographical locations. Raven and coworkers have recently postulated^30^ that the doubling times of photosynthetic organisms are directly correlated with their genome sizes. Our findings challenge this hypothesis, since the genome sizes of *Synechococcus* UTEX 2973 and *Synechococcus* PCC 7942 are nearly identical (Table 2), whereas their doubling times are significantly different. The large deletion region in the *Synechococcus* UTEX 2973 genome compared to the genomes of *Synechococcus* PCC 7942 and *Synechococcus* PCC 6301 presents an intriguing avenue for further study. Six genes present in this region are constitutively transcribed in *Synechococcus* PCC 7942. Most notably, genes that contained SNPs, indels and that are within the missing region in *Synechococcus* UTEX 2973 could be determinants of the growth rates of the cells.

In this study, we have generated global proteomics data for both *Synechococcus* UTEX 2973 and PCC 7942. These data support the genome sequencing data by showing corresponding amino acid differences between the two strains that were predicted by nucleotide sequence comparison (Table 3). Moreover, *Synechococcus* PCC 7942 is a widely used model cyanobacterial strain; however, its global proteome has not been analyzed thus far. Previous proteomic analysis based on 2D-PAGE identified a small fraction (~2%) of the total predicted proteome of *Synechococcus* PCC 7942^31^. Our global proteomic data with a coverage of 68% would be a valuable resource for the research community.

We have reported the occurrence of many electron-dense bodies in *Synechococcus* PCC 7942 under 3% CO_2_ conditions, and the absence of these bodies under the same conditions in *Synechococcus* UTEX 2973. Although the exact nature of these bodies is uncertain, their shape, size and number are similar to the glycogen granules that have previously been identified in *Synechococcus* PCC 7942^32^. However, in the previous study, glycogen granules were only visualized as electron-dense particles after treatment with a polysaccharide stain, whereas under our conditions these granules appear electron dense with standard treatment. If these are glycogen stores, the lack of these bodies in *Synechococcus* UTEX 2973 under high CO_2_ conditions might suggest fundamental differences in carbon utilization in these two strains. We hypothesize that *Synechococcus* UTEX 2973 may channel fixed carbon into biomass for rapid growth, while *Synechococcus* PCC 7942 stores carbon as glycogen.

Culture conditions that are optimal for the rapid growth of *Synechococcus* UTEX 2973 (38–41°C, 3% CO_2_ and 500 µmole photons·m^−2^·s^−1^ using BG11 media) are utilized in many laboratories, and no special nutrients, such as vitamins, are required for the growth of this cyanobacterial strain. This strain grows so rapidly, in culture volumes ranging from 50 ml to 100 L, such as in a photobioreactor, that contamination was not apparent even when growth media were not sterilized and the systems were semi-open to the outside. On solid BG11 plates, at 38°C and under 200 μmoles photons·m^−2^·s^−1^ light and in ambient air, single colonies of *Synechococcus* UTEX 2973 were visible within 2 days after plating from a very dilute liquid culture, and these colonies were large enough for passage by the third day. Such reduced colony formation time is immensely beneficial for genetic manipulation studies. Furthermore, in the *nblA* deletion experiment, transformed cells from a single colony were completely segregated in the first round after patching. The same result was observed in a subsequent experiment, where a foreign gene was inserted into the chromosome of *Synechococcus* UTEX 2973. Therefore, the elapsed time from conjugation to segregated mutant was approximately 8 days for this strain. In our experience, the same process in *Synechocystis* 6803 (~8 days or more for colonies of mutants to show^16^, plus several rounds of segregation) usually takes nearly one month. Expression of EYFP and inactivation of the *nblA* gene were two of many genetic manipulations (data not shown) that we have performed with *Synechococcus* UTEX 2973. In addition, we have determined that antibiotic resistance genes, including those conferring kanamycin resistance (*Tn903*), chloramphenicol resistance (*cat*), gentamicin resistance (*accC1*) and spectinomycin/streptomycin resistance (omega cassette), that have been used in other model cyanobacteria, also function in *Synechococcus* UTEX 2973.

According to our observations, *Synechococcus* UTEX 2973, like *Synechococcus* PCC 6301, is not naturally competent for transformation. This is unlike the naturally competent *Synechococcus* PCC 7942. Porin-like sequences near the edges of the large genomic inversion region (Fig. 2) have been hypothesized to be responsible for such natural transformability^20^. *Synechococcus* UTEX 2973 has the same genome structure as *Synechococcus* PCC 6301 on that inversion. According to this hypothesis, it would be possible to make *Synechococcus* UTEX 2973 naturally competent by flipping the inverted region on the genome. However, that is not necessary since conjugation requires limited extra time, and in some instances, conjugation may be the preferred route of DNA transfer, particularly when a large segment of foreign DNA is to be transferred or if merodiploids (single recombination events) are desired^26^. With attributes including rapid growth, amenability to genetic manipulation, and fast segregation, *Synechococcus* UTEX 2973 is expected to serve as an ideal cyanobacterial system for a wide range of applications.

## Methods

### Cyanobacterial strains and growth conditions

The Pakrasi lab procured the *Synechococcus elongatus* UTEX 625 strain from the UTEX algae culture collection (www.utex.org). *Synechococcus* 625 was not axenic when it was frozen and stored at −80°C in 2008. In addition, it could not grow at 38°C (see Results section). The frozen sample of *Synechococcus* UTEX 625 was thawed in 2011 and a single colony from the recovered culture, grown at 38°C, was picked and propagated. Cultures from this single colony were unialgal and no bacterial contamination was found when tested on LB medium. In addition, this strain could grow fast under photoautotrophic condition (see Results section). This *clean* strain was deposited to the UTEX algae culture collection, and a new number, UTEX 2973, was assigned to it. *Synechococcus elongatus* PCC 6301 and PCC 7942 were procured from the Pasteur Culture Collection of Cyanobacteria (PCC). Except when otherwise indicated, these strains were maintained in liquid BG11 or on solid BG11 plates^19^ at 38°C under continuous white light (70 µmole photons·m^−2^·s^−1^). *Synechococcus* sp. PCC 7002 was obtained from Prof. Toivo Kallas (University of Wisconsin, Oshkosh) and was grown at 38°C. *Synechocystis* sp. PCC 6803 was from the Pakrasi lab and was grown at 30°C. Growth under continuous light was measured as OD_730nm_ on a Multi-cultivator MC 1000-OD (Photon Systems Instruments, Drasov, Czech Republic), and growth under sine wave illumination, modulated to simulate natural sunlight, was measured on an FMT-150 photobioreactor as OD_735_^33^ (Photon Systems Instruments, Drasov, Czech Republic). CO_2_ gas flow rate at 1 ml/min and 3% (v/v) to every ml of culture was precisely controlled by a custom mixing system (Qubit Systems Inc, Kingston, Ontario). For doubling time measurements, five sets of measurements were made for each strain. Points were plotted as semi-log graphs and the specific growth rate K’ during the early exponential phase for each measurement was determined. The doubling time was then calculated as Ln2/K’. For sine wave illumination growth experiments, the mean value of OD_735 _at each time point was plotted. Blue and red light intensities were both set to change from 0 µmole photons·m^−2^·s^−1^ at 0 h to 250 µmole photons·m^−2^·s^−1^ at 6 h, and then decrease to 0 at 12 h. Red light increased more steeply during morning and evening hours than blue light. This setting of the photobioreactors is based on the description in ref. 33. For large scale (100 L culture volumes) growth, algal photobioreactors (Photon Systems Instruments, Drasov, CZ) in the Advanced Coal & Energy Research Facility at Washington University in St. Louis (http://cccu.wustl.edu/research-facil.php) were used. Biomass of cyanobacterial cultures was determined using a method slightly modified from that described previously^34^.

### Whole genome sequencing

Illumina genome sequencing was performed at the Genome Technology Access Center (GTAC) at Washington University. Genomic DNA was isolated and sonicated to an average size of 175 bp. The DNA fragments were repaired to produce blunt ends, modified with a 3’ ‘A’ base overhang, and ligated to Illumina's standard sequencing adapters. The ligated fragments were amplified for 10 cycles incorporating a unique indexing sequence tag. The resulting libraries were sequenced as 101 nucleotide paired end reads using the Illumina HiSeq-2000. Sequence reads were mapped to *Synechococcus* PCC 6301 and *Synechococcus* PCC 7942 genome data obtained from GenBank (NC_006576 and NC_007604, respectively) using Novoalign. SAMtools was used to identify SNPs between the two strains. Dindel was used to identify indels. Pindel and custom analyses were used to identify structural rearrangements. 454 genome sequencing was also performed in parallel (MOgene LC, St. Louis, MO) and the genome was assembled *de novo* using Newbler 2.8.

Assembled chromosome and two plasmid sequences of *Synechococcus* UTEX 2973 were deposited to Genbank (Accession number: CP006471 to CP006473).

### Sample preparation for Proteomics and LC-MS/MS analysis

Cell suspensions of both *Synechococcus* UTEX 2973 and *Synechococcus* PCC 7942 were disrupted and digested into tryptic peptide form as previously described^35^,^36^. The resulting isolated peptide samples were separated via a high pH HPLC approach (Agilent 1200 HPLC System) as previously described^37^, resulting in the automated collection of 96 fractions, which were lyophilized and reconstituted into 24 fractions prior to LC-MS/MS analysis. Fractionated samples were subjected to LC-MS/MS analysis using a Thermo Scientific LTQ mass spectrometer (Thermo Scientific, San Jose, CA, USA) coupled with an in-house electrospray ionization interface as previously described^38^,^39^,^40^. LC–MS/MS raw data were converted into data files using Bioworks Cluster 3.2 (Thermo Fisher Scientific, Cambridge, MA, USA), and the MSGF+ algorithm was used to search MS/MS spectra against *Synechococcus* UTEX 2973 and *Synechococcus* PCC 7942 databases (NCBI). Key search parameters included 20 ppm tolerance for precursor ion masses, partial tryptic search, with decoy database searching methodology^41^ used to control the false discovery rate at the unique peptide level to ~0.1%^42^. Global proteomics data for both *Synechococcus* UTEX 2973 and *Synechococcus* PCC 7942 have been uploaded to PeptideAtlas with the dataset identifier number of PASS00399.

### Electron microscopy

Cells were prepared for electron microscopy as described previously^43^. Culture aliquots (approximately 20 ml) were centrifuged, and the pellet was resuspended in a small volume, transferred to planchettes with 100–200 µm-deep wells, and frozen in a Baltec High Pressure Freezer (Bal-Tec). Samples were freeze-substituted in 2% osmium/acetone (3 d at −80°C, 15 h at −60°C, slow thaw to room temperature) and embedded in Spurr's resin. Thin sections (approximately 80 nm) were stained with uranyl acetate and lead citrate. Digital images were viewed and collected using a LEO 912 transmission electron microscope operating at 120 kV and a ProScan digital camera.

### Genetic modification of *Synechococcus* UTEX 2973

Tri-parental conjugation was used to generate mutants of *Synechococcus* UTEX 2973^22^, with pRL443 as the conjugal plasmid and pRL623 as the helper plasmid^44^. Shuttle or suicide vectors carrying the gene of interest were first transformed into competent HB101 *E. coli* cells that already contained the pRL623 helper plasmid to form cargo strains. 100 μl overnight cultures of the conjugal strain (RL443) and 100 μl overnight cultures of the cargo strain were washed with distilled water and mixed with pre-washed *Synechococcus* UTEX 2973 cells (200 μl at OD_730_ ~0.4-0.6). Mixed cells were plated onto BG11+5% LB (v/v) agar plates containing selective antibiotics. Plates were placed under bright continuous illumination (200 μmole photons·m^−2^·s^−1^). Mutant colonies were usually apparent within 3–4 days. The shuttle vector pTrc-EYFP for eYFP expression is a derivative of pPMQAK1-BBa_P1010^15^. The suicide plasmid pSL2231 used to delete the *nblA* gene in *Synechococcus* UTEX 2973 was a derivative of pBR322. The coding region of the *nblA* gene was targeted for deletion from the chromosome after homologous recombination. 581 bp upstream and 558 bp downstream of *nblA* coding region were amplified using primers N1: ATATAAGCTTGATGCGCAGATAGCCTGACTGTTCC/N2: CCAGGATTGGGAGGCTCCGGCACTGCAGATGAAC; and P1: TCGACTCTACCGTGTGCAAGACTTGCCCGCGAAG/P2: ATATGGATCCATGCTGCTGGAGTTCTACGCCGAC, respectively. The upstream and downstream sequences were fused with a chloramphenicol resistance gene (Cm^R^, *cat*, amplified using primers O1: AGCCTCCCAATCCTGGTGTCCCTGTTGATACC/O2: CACACGGTAGAGTCGACGAATTTCTGCCATTCATCC) by fusion PCR to form an upstream- Cm^R^ -downstream fragment. This fragment was inserted into the pBR322 vector between *Hind*III and *Bam*HI sites to form the plasmid pSL2231 (Figure S2).

### Fluorescence microscopy

Concentrated cells of WT and EYFP mutant strains from 2-day old cultures were deposited onto glass slides that were previously coated with 2% polyethyleneimine. Cells were imaged using a Nikon Eclipse 80i microscope equipped with a Photometrics Cool Snap ES CCD camera (Roper Scientific). Illumination was provided by a metal halide light source (X-Cite). Filter sets (Chroma) were as follows: YFP was detected using a 480/30 nm excitation filter, a 505 nm dichroic beam splitter, and a 535/40 nm emission filter. Chlorophyll fluorescence was detected using a 560/40 nm excitation filter, a 595 nm dichroic beam splitter, and a 630/60 nm emission filter. A 300 ms exposure time was used for imaging.

### Whole cell absorbance spectra

These spectra were obtained using a μQuant plate reader (Bio-Tek Instruments, Winooski, VT). 150 μl culture volumes in 96-well plates were measured from 400 nm to 800 nm. Absorption curves were vertically offset (indicated by “relative OD”) for comparison purposes.

## Supplementary Material

Supplementary Information Dataset 1

## Figures and Tables

**Table 1:** Doubling times of *Synechococcus* UTEX 2973 and other model cyanobacterial strains under different conditions. The colors of highlighted values correspond to the colors of the growth curves shown in Fig 1

	Temp (°C)	41	38						30
	CO_2_ (v/v)	3%	3%				Air (0.04%)		3%
	Light intensity*	500	100	200	300	500	100	500	300
UTEX 2973	Doubling time (hrs)	**2.1 ± 0.2**	5.0 ± 0.4	-	4.2 ± 0.3	2.3 ± 0.1	6.3 ± 1.7	5.4 ± 0.9	-
PCC 7942		-	6.1 ± 0.3	-	**4.9 ± 0.7**	8.5 ± 0.3	8.7 ± 0.7	-	-
PCC 7002		-	-	9.3 ± 0.3	-	**4.1 ± 0.4**	-	-	-
PCC 6301		-	-	-	-	**5.4 ± 0.3**	-	-	-
PCC 6803		NG	NG	NG	NG	NG	NG	NG	**6.6**

Means +/− standard deviation of 5 replicates, except for *Synechocystis* PCC 6803, for which the mean doubling time of two replicates is shown.

*, in µmole photons·m^−2^·s^−1^.

-, not determined.

NG, no growth.

**Table 2:** General features of the genome of *Synechococcus* UTEX 2973 compared to genomes of other related cyanobacteria

	*Synechococcus* UTEX 2973*	*Synechococcus* PCC 7942*	*Synechococcus* PCC 6301*
Genome size (bp)	2,690,418	2,695,903	2,696,255
GC content	55.4%	55.4%	55.5%
Protein coding genes	2,645	2,661	2,525
rRNA operons	6	6	6
tRNA genes	44	44	45

*, data from NCBI Genbank and JGI database.

**Table 3:** SNPs and indels in coding regions of *Synechococcus* UTEX 2973 chromosome (accession number in NCBI: CP_006471) compared to the reference genome of *Synechococcus* PCC 7942 (CP_000100). Corresponding differences in amino acids in the encoded proteins in *Synechococcus* UTEX 2973 are shown. RFC, Reading Frame Change

Nucleotide position in UTEX 2973 genome	Locus tag (M744)	Protein	Amino acid change	Comments
126938	00705	Hypothetical protein	STOP 146 L	159 aa in PCC 7942
236706	01335	ATP synthase F_0_F_1_ subunit alpha	Y 252 C	Confirmed by proteomics data
475352	02605	Chemotaxis protein CheY	R 121 Q	
475390	02605	Chemotaxis protein CheY	K 134 E	
518500	02855	Pili assembly chaperone	A 52 D	
606448	03320	23S ribosomal RNA		
608335	03320	23S ribosomal RNA		
610804	03335	Manganese ABC transporter ATP-binding protein	D 225 H	
709129	03855	Guanylate cyclase	C 35 R	Annotated as serine phosphatase in PCC 7942
731334-731576	03965	ABC-transporter substrate-binding protein	Deletion of 243 bp	Annotated as adenine phosphoribosytransferase in PCC 7942
733938	03975	Hypothetical protein	STOP 113 Q	238 aa PilN-like protein in PCC 7942, confirmed by proteomics data
891346	04780	PpnK, inorganic polyphosphate/ATP-NAD kinase	D 260 E	
1080351	05865	Hypothetical protein	L 133 R (RFC)	135 aa in UTEX 2973134 aa in PCC 7942
1113358	06025	Molecular chaperone DnaK	G 196 S	
1222741	06570	Hydrolase	F 165 V	Annotated as peptidase M20D, amidohydrolase in PCC 7942
1222972	06570	Hydrolase	R 247 G (RFC)	395 aa in UTEX 2973252 aa in PCC 7942
1237531	06650	CTP synthetase	A 294 V	
1238113	06650	CTP synthetase	R 488 H	
1273424	06850	Chorismate mutase	G 122 V (RFC)	139 aa in UTEX 2973130 aa in 7942
1619501	08615	DNA-directed RNA polymerase β subunit	P 378 Q	Confirmed by proteomics data
1718274	09095	Porin; major outer membrane protein	Silent	
2249045	11685	Anthranilate synthase, component I	V 329 G	
2339739	12130	Long-chain-fatty-acid CoA ligase	R 216 P	Confirmed by proteomics data
2339502	12130	Long-chain-fatty-acid CoA ligase	P 295 L	
2364720	12285	Glutamate synthase	L 1018 S	Confirmed by proteomics data
2608863	13540	Photosystem I assembly protein Ycf4	F 39 L	

**Table 4:** SNPs and indels in the coding regions in the small plasmid of *Synechococcus* UTEX 2973 (CP_006473) compared to those in the small plasmid pUH24 in *Synechococcus* PCC 7942 (NC_004990). Corresponding amino acid changes in the encoded proteins are shown. RFC, Reading Frame Change

Nucleotide position in UTEX 2973 small plasmid	Locus tag (M744)	Protein	Amino acid changes	Comments
108		Not an ORF		Hypothetical protein in PCC 7942
1038	14245	Hypothetical protein	E 158 D	
1039	14245	Hypothetical protein	L 159 V	
2460	14250	Hypothetical protein	A 841 V (RFC)	1019 aa in UTEX 2973876 aa in PCC 7942
4039	14250	Hypothetical protein	K 314 N	
4040	14250	Hypothetical protein	Q 315 E	
6809	14260	Hypothetical protein	A 175 V (RFC)	177 aa in UTEX 2973187 aa in PCC 7942
