# Supplementary material for: Synechococcus elongatus UTEX 2973, a fast growing cyanobacterial chassis for biosynthesis using light and CO2
Source: Sci Rep. 2015 Jan 30;5:8132. doi: 10.1038/srep08132 (PMC5389031; doi:10.1038/srep08132)
Supplement: Supplementary Information — Dataset 1 [file srep08132-s1.pdf]

Synechococcus elongatus UTEX 2973, a fast growing cyanobacterial chassis for biosynthesis using light and CO_2

Jingjie Yu[1], Michelle Liberton[1], Paul F. Cliften[2], Richard D. Head[2], Jon M. Jacobs[3], Richard D. Smith[3], David W. Koppenaal[3], Jerry J. Brand[4] & Himadri B. Pakrasi[1,*]

[1]Department of Biology, Washington University, St. Louis, MO 63130

[2]Genome Technology Access Center, Washington University School of Medicine, St. Louis, MO 63110

[3]Pacific Northwest National Laboratory, Richland, WA 99352

[4]UTEX The Culture Collection of Algae, University of Texas, Austin, TX 78712

*Correspondence and request for materials should be addressed to H.B.P.

Phone – 314-935-6853; Fax – 314-935-8818; Email - pakrasi@wustl.edu

Table S1. ORFs in the *Synechococcus* 7942 genome that are missing in the *Synechococcus* 2973 genome

Locus tag (Synpcc7942)	Nucleotide location	protein	Notes
0767	759413-759847	Hypothetical protein	
0768	760522-761178	Hypothetical protein	confirmed by proteomics data (19 unique peptides)
0769	761226-762128	Hypothetical protein	confirmed by proteomics data (5 unique peptides)
0770	762159-763643	Hypothetical protein	confirmed by proteomics data (25 unique peptides)
0771	763640-764854	Hypothetical protein	confirmed by proteomics data (5 unique peptides)
0772	764984-765577	Chromophore Lyase CpcT/CpeT	confirmed by proteomics data (4 unique peptides)

Table S2. Peptide sequence comparisons of genes on the edges of the region of inversion between UTEX 2973 and PCC 7942. Dots represent the amino acids that are identical to the corresponding ones in the middle line.

Edge 1 peptide alignment

Upper line: UTEX 2973 M744_08310
Middle line: Amino acid sequence
Bottom line: PCC 7942 synpcc7942_1463

```
         10        20        30        40        50        60        70        80        90
....|....|....|....|....|....|....|....|....|....|....|....|....|....|....|....|....|....|
MKNLFKVMLAGPLAASVAFAGAAQAESLNRDTINQVNAIANEELPAAQVTSITQLSDVKPTDWAYQALQSLVERYGCIVGYPDRTYRGSR
----MH.PA.LWMGTGLWLGL.PATIAFDAS..R........D...............R...............................

        100       110       120       130       140       150       160       170       180
....|....|....|....|....|....|....|....|....|....|....|....|....|....|....|....|....|....|
ALTRYEFAAGLNACLDKVIEFAASKEDLDTLKKLMEEFQAELATLRGRVDSLEARVTELEATQFSTTTKLQGEVILSLDRAFGPSGLDTG
..........................................................................................

        190       200       210       220       230       240       250       260       270
....|....|....|....|....|....|....|....|....|....|....|....|....|....|....|....|....|....|
TSFSQRVSLNLNTSFTGKDLLKTRLRSNSITSPGLRDFNGGVPGSGIISPAAALDYDNATNAPNANFFLDTLLYTFPVGDVKFTVGTSNL
..........................................................................................

        280       290       300       310       320       330       340       350       360
....|....|....|....|....|....|....|....|....|....|....|....|....|....|....|....|....|....|
QVEDILSTTGTFYAPYISYFFSTPIPGIYGDADTTNSAGAGFNWQLSKNFNFGAAYINQNGPTTGGNLASDPTSGVFGADSQTTAQLAFK
..........................................................................................

        370       380       390       400       410       420       430       440       450
....|....|....|....|....|....|....|....|....|....|....|....|....|....|....|....|....|....|
NDSGTFIGALAYAYRKGPDLATDLSFLPSQFGGVNFGTPRALAGLGLGSSGSDIVSTNNIGLSLGWAVSDGFTISGSYGISFGNGFGANN
..........................................................................................

        460       470       480       490       500       510       520       530       540
....|....|....|....|....|....|....|....|....|....|....|....|....|....|....|....|....|....|
TVQSWTVGFTFPNLFADGNEFGFAVGQAPYVISDSRGAAFQDSGNFAFEVYYKLQVTDNISITPALYAITNAGGGLSFPDSANGDFIVPV
..........................................................................................


....|..
IKTVFTF
.......
```

Edge 2 peptide alignment

Upper line: UTEX 2973 M744_09255
Middle line: Amino acid sequence
Bottom line: PCC 7942 synpcc7942_1635

```
         10        20        30        40        50        60        70        80        90
....|....|....|....|....|....|....|....|....|....|....|....|....|....|....|....|....|....|
MS--MHVPAALWMGTGLWLGLAPATIAFDASTIRQVNAIANEDLPAAQVTSITQLSDVRPTDWAYQALQSLVERYGCIVGYPDRTYRGSR
.KNLFK.ML.GPLAASVAFAG.AQAESLNRD..N........E...............K...............................

        100       110       120       130       140       150       160       170       180
....|....|....|....|....|....|....|....|....|....|....|....|....|....|....|....|....|....|
ALTRYEFAAGLNACLDKVIEFAASKEDLDTLKKLTEEFQSELATLRGRVDGLEARVTELEATQFSTTTKLQGDVLFSLDRAFGPSGLDTG
..........................................................................................

        190       200       210       220       230       240       250       260       270
```

```
....|....|....|....|....|....|....|....|....|....|....|....|....|....|....|....|....|....|
TSFSQRVGLDFNTSFTGKDLLKTRLETNSITSPGLRDLNGFAAGAGIPSPGAALAYDNAATGADPNFTLNTLIYQFPVGSVNFTVGTSNV
..........................................................................................

        280       290       300       310       320       330       340       350       360
....|....|....|....|....|....|....|....|....|....|....|....|....|....|....|....|....|....|
QVDDVFSTNGSFYAPELSYFFNNPVPGIYNDADTSNSAGAGFNWQINPNFNLGLAYINQQGPTSGQNLEADPTAGVFGADSQTTAQLAFK
..........................................................................................

        370       380       390       400       410       420       430       440       450
....|....|....|....|....|....|....|....|....|....|....|....|....|....|....|....|....|....|
NDDGTFIGALAYAYRKGPAFDPVNPDGVGLPAQFGGVSYGTVRSLIGPSNSPTDLISSNNIGLSLGWAVSENFTISGSYGISFLSGSAGS
..........................................................................................

        460       470       480       490       500       510       520       530       540
....|....|....|....|....|....|....|....|....|....|....|....|....|....|....|....|....|....|
STVQSWNIGLTFPNLFADGNELGLAIGQIPYVTSDSRGAAFADSGPFAFEVYYKLQLTDNIAITPALYAVTNAGGGLTLGNLANGDYWVP
..........................................................................................

....|...
VLKTEFLF
........
```

Figure S1.

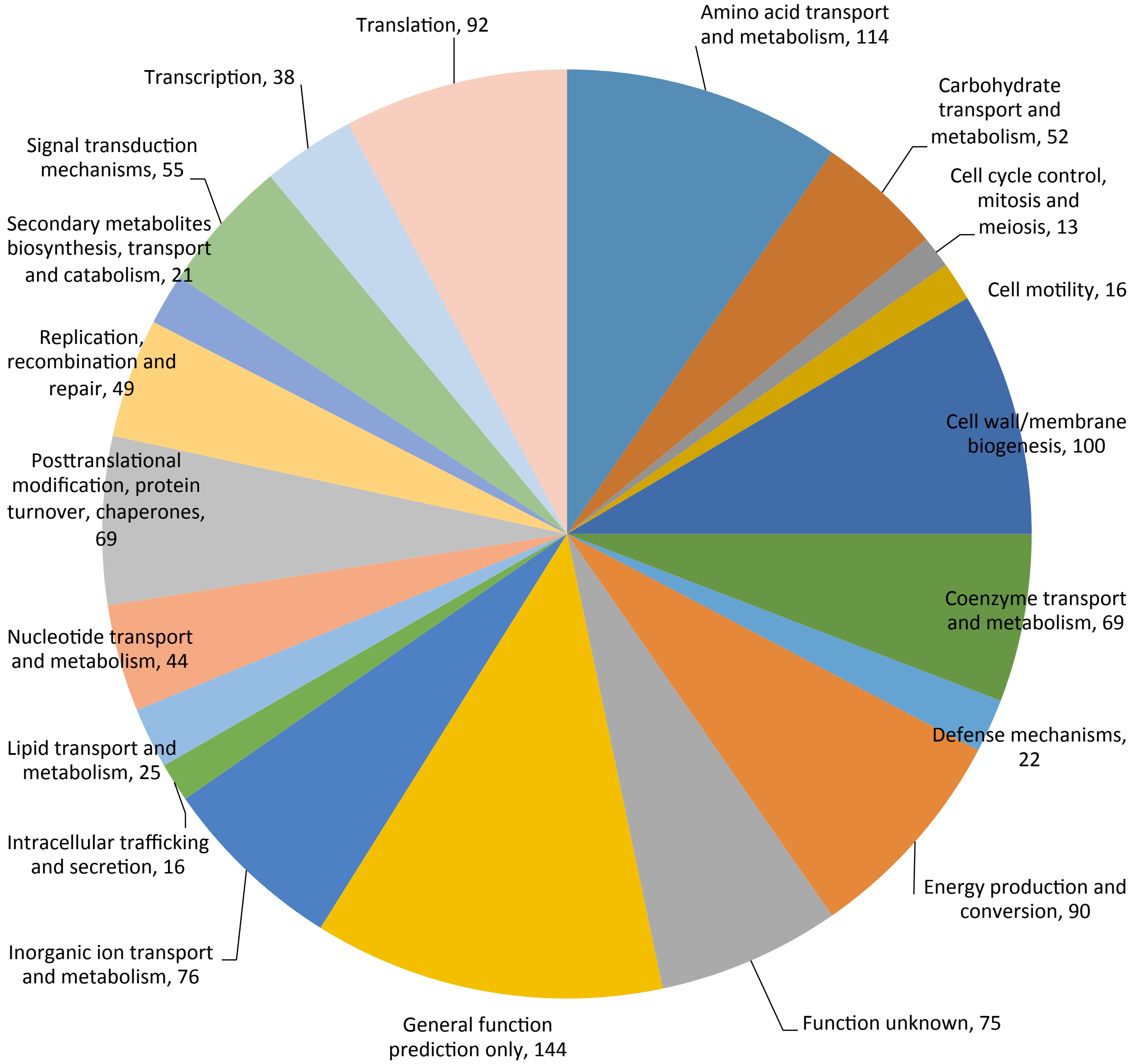

Figure S1. Categories of functional proteins of UTEX 2973 detected in global protein mass spectra.

Figure S2.

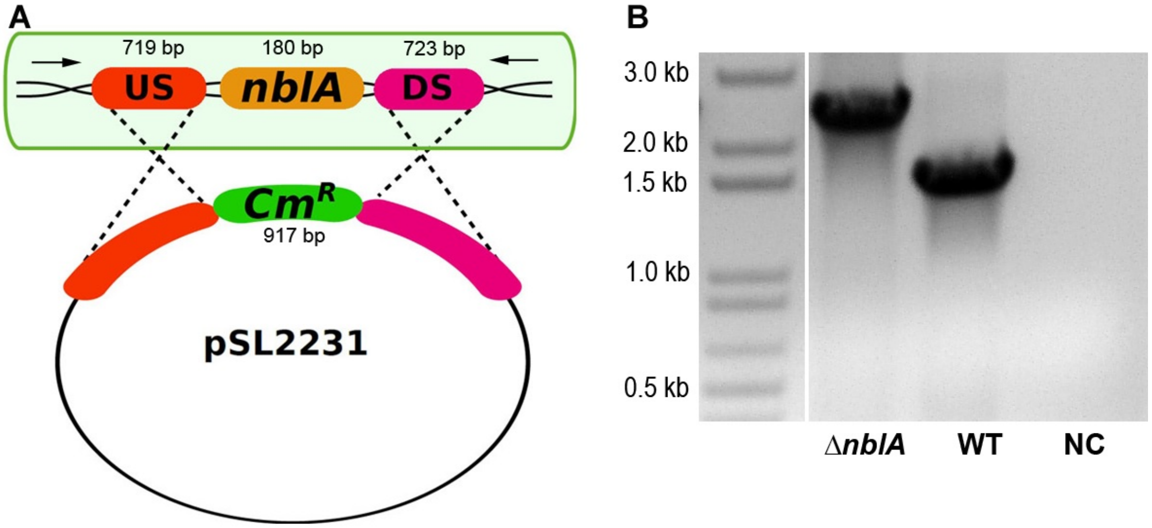

Figure S2. Verification of gene replacement in the *Synechococcus* UTEX 2973 Δ*nblA* mutant strain. A. Expected double reciprocal homologous recombination. US: upstream sequence; DS: downstream sequence; Cm^R: chloramphenicol resistance gene cassette; pSL2231: a plasmid derivative of pBR322. Sizes of all fragments are indicated. Arrows represent primers used to verify recombination results. **B**. PCR products from Δ*nblA* mutant and WT cells using primers shown in (A). The expected size of the PCR product from WT cells is 1622 bp; while the expected size of the PCR product from the Δ*nblA* mutant with the coding region of *nblA* replaced by Cm^R gene is 2359 bp. Absence of the 1.6 kb WT band in Δ*nblA* lane indicated that the mutant strain had completely segregated. NC: negative control (without template).
